# Risk for Postmarket Black Box Warnings in FDA-Approved Monoclonal Antibodies

**DOI:** 10.1016/j.mayocpiqo.2021.11.009

**Published:** 2021-12-28

**Authors:** John B. Hagan, Elizabeth Ender, Rohit D. Divekar, Thanai Pongdee, Matthew A. Rank

**Affiliations:** a Division of Allergic Diseases, Mayo Clinic, Rochester, MN; b Internal Medicine-Pediatrics, Marshfield Clinic, Marshfield, WI; c Division of Allergy, Asthma, and Clinical Immunology, Mayo Clinic, Scottsdale, AZ

**Keywords:** BBW, black box warning, FDA, US Food and Drug Administration, IQR, interquartile range, mAb, monoclonal antibody, NA, not applicable, PML, progressive multifocal leukoencephalopathy

## Abstract

**Objective:**

To estimate the potential risk for a future postmarket black box warning (BBW) of US Food and Drug Administration (FDA)-approved monoclonal antibodies (mAbs) because of the importance for medical clinicians to understand mAb risks and benefits, including unknown future risks, especially for recently approved mAbs.

**Methods:**

The complete dates of the study were March 16, 2020, through May 12, 2021. We searched the FDALabel database online and reviewed the scientific literature to determine current and previous FDA-approved mAbs as of March 2020. The BBWs and initial FDA-issued safety warnings were identified. The BBWs were categorized as premarket or postmarket. For mAbs with specific postmarket BBWs, previous FDA labels were evaluated to identify the presence or absence of an initial corresponding specific FDA warning.

**Results:**

In March 2020, a total of 83 mAbs had FDA approval; 33 had BBWs (27 premarket and 13 postmarket BBWs). Of these 33 mAbs, 55 individual specific BBWs existed (36 premarket and 19 postmarket specific warnings). On average, the specific BBWs occurred in the postmarket period at a rate of 3.4% (19/562) per year. Most (73.7%; 14/19) specific postmarket BBWs were preceded by an FDA warning in a median time of 3.61 (interquartile range, 1.36-5.78) years. Specific postmarket BBWs not preceded by a specific FDA product label warning occurred at an average rate of 0.9% (5/562) per year.

**Conclusion:**

Specific postmarket BBWs occurred in FDA-approved mAbs at a rate of 3.4% per year. Specific postmarket BBWs not preceded by a specific FDA product label warning had a rate of 0.9% per year.

In March 2020, more than 80 novel therapeutic monoclonal antibodies (mAbs) were actively on the market for human use, with more than 50% of these agents approved by the US Food and Drug Administration (FDA) within the past 5 years.^1^ This proliferation of novel agents has been associated with considerable anticipation of improved health-related outcomes for various medical conditions. Clinicians rely on the knowledge, expertise, and information provided by the FDA, which performs regulatory assessments that lead to the approval and postmarket safety analysis of mAbs.^2^

The FDA information about the potential risks, benefits, and limitations of agents is essential for clinicians who seek to optimally allocate treatments for their patients. The black box warning (BBW) is intended to denote the serious or life-threatening risks of an approved pharmacologic agent.^3^ A black box may contain 1 or more specific warnings. These warnings may be present at licensure and available for review as a serious potential risk before initiation of therapy. Alternatively, the warnings may be added, usually because of postmarket analysis, at a date after drug approval. In the latter case, the importance of the risk may not be available for risk and benefit review by the medical provider and the patient before initiation of the FDA-approved therapy. We sought to evaluate the potential risk for emerging BBWs, specific BBWs, and non–black box safety warnings occurring after initiation of therapy with an FDA-approved mAb.

## Methods

### Study Sample

To identify all therapeutic mAbs previously approved by the FDA, we performed a search of the FDALabel database^4^ for currently available lists of biological agents from the FDA’s Center for Biologics Evaluation and Research, Micromedex (IBM), and relevant literature, with a search date completion of March 27, 2020.^5^, ^6^, ^7^, ^8^, ^9^ The complete dates of the study were March 16, 2020, through May 12, 2021. Muromonab-CD3, daclizumab, and efalizumab were previously withdrawn from the market for safety concerns, and vedolizumab was withdrawn from the market voluntarily. These mAbs were not active in March 2020 and were also excluded from this study.

### Therapeutic Areas

The initial indication for FDA approval was determined by review of the initial approval letter at Drugs@FDA: FDA-Approved Drugs.^10^ This indication was used to classify each mAb into 1 of 10 therapeutic categories: cancer and hematology, autoimmune, dermatology, infectious disease, neurology, hyperlipidemia, musculoskeletal, cardiovascular, genitourinary and renal, and other.

### Special Regulatory Pathways

The FDA has provided regulatory pathways that allow for prioritization of the assessment process.^11^ The investigative team used the FDA-provided information as methodology to define various types of priority review used for mAbs. In 1992, priority review was permissible with use of the Prescription Drug User Fee Act that allowed for a 2-tier system of review times, wherein the standard 10-month review could be reduced to a 6-month priority review. The program was established for pharmacologic agents that had substantial improvement in safety or effectiveness or improved the prevention of serious conditions. Applicants could request priority review according to a specific protocol, which included a fee. The FDA made its decision about the priority status within 60 days of submission for biological licensing application or new drug application. The agency did not grant a change in trial times or an alteration of standards for drug approval or quality of evidence.

Accelerated approval was also allowed in 1992. It provided expedited review and approval for serious conditions based on a surrogate end point rather than a specific clinical outcome. In 2012, the accelerated approval also allowed for an intermediate clinical end point that could lead to agent approval. Accelerated approval in 1992 and 2012 was contingent on clinical outcome confirmation in postmarket analysis. Otherwise, the drug was to be withdrawn from the market. The drug sponsor was to submit and the FDA was to decide about an accelerated approval on the basis of criteria, such as scientific merit of the end point.

A fast-track process was established in 1997 to provide early and frequent communication between the sponsor and the FDA, thereby to more efficiently bring new agents for serious conditions to patients. Fast track could be requested by the sponsor anytime during the approval process, at which point the FDA would decide on the fast-track status within 60 days.

The breakthrough therapy application was established in 2012. This process was to attain expedited review of agents to treat a severe condition for which preliminary data showed clear benefit for serious outcomes such as morbidity and death or serious consequences of the disease process. The sponsor could submit for a breakthrough therapy designation. Alternatively, the FDA could suggest that the sponsor submit a request no later than the end of the phase 2 meeting. The FDA decision was to be made within 60 days.

The initial approval letters at the Drugs@FDA: FDA-Approved Drugs database were reviewed to determine whether mAbs were evaluated with use of priority review, accelerated approval, fast track, or breakthrough therapy.^10^

### Orphan Drug Status

Orphan drug designation began with the Orphan Drug Act of 1983. It uses such criteria as the mechanism of proposed treatment, disease intended to treat or prevent, disease pathogenesis, course of disease, and resistance to treatment whereby the orphan drug designation was established. Which mAbs achieved orphan status were determined with review of the Orphan Drug Product designation database.^12^

### Regulatory Review Times

The FDALabel and Drugs@FDA: FDA-Approved Drugs databases were used to determine the initial date of submission to the FDA and the day of FDA approval (ie, market date). The total review was calculated on the basis of the submission to the FDA until the drug received FDA approval. The total review time of an individual mAb was classified into prespecified categories.

### Postmarket Safety Events

The primary objective in this study was to determine the frequency and timing of BBWs that occur after FDA approval (ie, postmarket BBWs). The FDA databases such as Drugs@FDA: FDA-Approved Drugs and DailyMed (dailymed.nlm.nih.gov/dailymed/) were used to identify the presence or absence of BBWs on all previously approved mAbs.

It is important to recognize that pharmacologic agents such as mAbs may have a BBW denoting that the specific agent has 1 or more warnings within a black box. However, a black box with 1 warning is different than a black box with 2 or more warnings. We sought to distinguish these important differences by using the terminology "BBW" to denote an mAb having 1 or more warnings in a black box and "specific BBW," which refers to each specific warning in the black box.

Warnings were categorized as present at FDA approval or emerging in the postapproval period. We documented the number of mAbs on the market as of March 27, 2020. The time that elapsed from FDA approval to the date of the end of data acquisition (March 27, 2020) was determined for each mAb, and the sum of days was used to yield the total years of therapeutic mAb on the market. The risk that an FDA-approved mAb may acquire an emerging BBW after approval was calculated with the formula [total emerging BBWs in the postapproval period/(sum of years on the market for all currently approved mAbs)] × 100. Specific variables that may have led to alteration from the standard approval process (eg, priority status or fast-track approval) were analyzed to determine a possible relationship between the presence or absence of an emerging BBW in the postapproval period.

We determined the time from product approval to BBW and the time and frequency of a preceding warning in the FDA label of emerging postapproval BBWs. Incremental box warnings were also assessed through review of additional databases (Table 1).^13^, ^14^, ^15^, ^16^ In addition to BBWs and specific BBWs, initial FDA-issued safety warnings (FDA warnings including those that may not appear in a black box) were identified and frequency, time to warning, and relation to special regulatory pathway were characterized.

**Table 1:** Incremental Box Warnings Assessed Through Additional FDA Databases

MedWatch, Safety Alerts, 1996-2016 http://wayback.archive-it.org/7993/20170110235327/http:/www.fda.gov/Safety/MedWatch/SafetyInformation/default.htm Completed January 1, 2017, through December 31, 1996^13^
Drug Safety Communications https://www.fda.gov/drugs/drug-safety-and-availability/drug-safety-communications^14^
Index to Drug-Specific Information https://www.fda.gov/drugs/postmarket-drug-safety-information-patients-and-providers/index-drug-specific-information^15^
Drug Safety-related Labeling Changes https://www.accessdata.fda.gov/scripts/cder/safetylabelingchanges^16^

FDA = US Food and Drug Administration.

### Warning Categories

Postapproval warnings were classified after review of the information into the following categories: reproductive, malignancy, infusion reaction, infectious disease, dermatology, and cardiovascular.

### Statistical Analyses

We used descriptive statistics to characterize the frequency and time course of safety events that occurred in association with FDA-approved mAbs. Central tendency of nonparametric data was reported as median with interquartile range (IQR). Pearson χ^2^ and Fisher exact tests were used to analyze categorical data. For nonparametric data, Wilcoxon/Kruskall-Wallis (rank sums) test was used for comparing groups. Statistical tests were 2 tailed, and *P*<.05 was the significance threshold for all comparisons. We used statistical software (JMP, version 14.1.0; SAS Institute Inc) to conduct all analyses.

## Results

**Figure 1:**
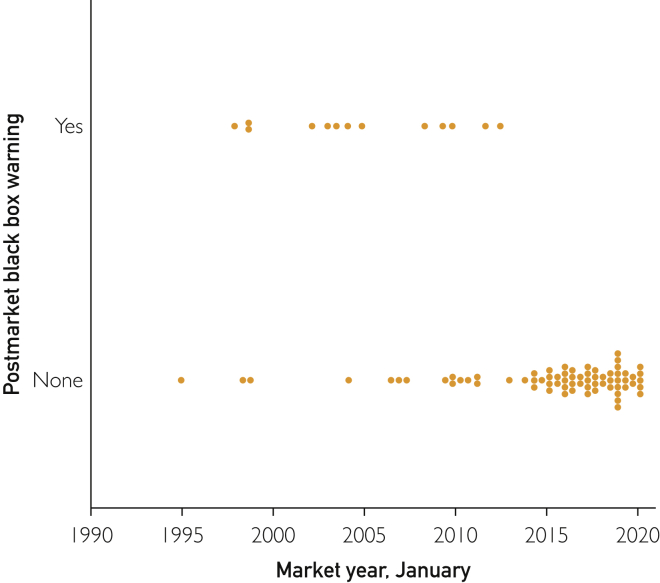
Initial postmarket black box warnings according to market year. Of the 83 monoclonal antibodies, 13 had a postmarket black box warning.

Through March 2020, the FDA had approved 87 specific mAbs for therapeutic use in humans, of which 83 were on the market (Table 2). Thirty-three mAbs (27 mAbs with preapproval and 13 mAbs with postapproval) had 1 or more BBWs. Postmarket BBWs were more likely to have occurred for FDA-approved mAbs that received approval before 2013 than mAbs approved between 2013 and March 2020 (Figure 1). The BBWs were not associated with priority review, accelerated approval, breakthrough therapy, fast-track approval, orphan drug status, premarket BBW, or time from FDA submission to approval.

**Table 2:** Therapeutic and FDA Status of 83 mAbs^a^

mAb Status	mAb, no. (%)
Therapeutic area	
Cancer and hematology	38 (45.8)
Autoimmune	11 (13.3)
Dermatology	8 (9.6)
Infectious disease	5 (6.0)
Neurology	5 (6.0)
Hyperlipidemia	2 (2.4)
Musculoskeletal	2 (2.4)
Cardiovascular	1 (1.2)
Genitourinary and renal	1 (1.2)
Other	10 (12.0)
Priority review^b^	
Yes	50 (60.2)
No	33 (39.8)
Accelerated approval	
Yes	19 (22.9)
No	64 (77.1)
Fast-track approval	
Yes	24 (28.9)
No	59 (71.1)
Breakthrough therapy	
Yes	32 (38.6)
No	51 (61.4)
Orphan drug	
Yes	40 (48.2)
No	43 (51.8)
Regulatory review time	
Total review time (d), median (IQR)	269 (189-365)
<200	24 (28.9)
200-399	46 (55.4)
≥400	13 (15.7)
Follow-up (y), median (IQR)	4.16 (1.94-9.06)

a FDA = US Food and Drug Administration; IQR = interquartile range; mAb = monoclonal antibody.

b Defined as either “Priority review/Prescription Drug User Fee Act <200 days” or “Negative.”

Of the 33 mAbs with BBWs, a total of 55 individual BBWs were issued (36 [65.5%] preapproval and 19 postapproval specific warnings). Of the 19 postapproval warnings, the most prevalent category was infectious disease, followed in frequency by malignancy, infusion reactions, dermatology, cardiovascular, and reproductive reactions.

The 83 mAbs currently on the market as of March 2020 encompass 562 years of approved mAb use. On average, a BBW emerged in the postapproval period at a rate of 3.4% (19/562) per year. Of the 19 emerging postmarket BBWs, 14 (73.7%) had been preceded by a safety warning that had been present in 1 or more previous FDA labels (median, 3.61; IQR, 1.36-5.78 years).

"Of 19 BBWs which occurred in the post market period, only 5 had no preceding warning (rate, 0.9% per year)." These mAbs were: (1) cetuximab on March 1, 2006, for cardiopulmonary arrest; (2) ibritumomab on September 13, 2005, for severe cutaneous mucocutaneous reactions; (3) natalizumab on June 5, 2006, for progressive multifocal leukoencephalopathy (PML); (4) rituximab on February 10, 2006, for severe mucocutaneous reactions; and (5) trastuzumab on December 11, 2001, for infusion reaction and pulmonary toxicity. Table 3 summarizes all mAbs currently approved that contain PML as a general warning in the Warning & Precautions Section of the product label or BBW.

**Table 3:** Currently Approved Monoclonal Antibodies With BBW or Warning and Precautions Section Caution for PML^a^

Monoclonal Antibody	BBW	Date of Warning	BBW vs Warning and Precaution	If Postmarket BBW, Previous Warning?	PML Risk
Natalizumab	Yes	6/5/2006	Postmarket BBW	No	PML known increased risk, cofactor risks identified and guidance
Rituximab	Yes	2/21/2007	Postmarket BBW	Yes	Fatal PML reported
Brentuximab	Yes	1/13/2012	Postmarket BBW	Yes	Fatal PML reported within 3 mo of initial exposure
Ofatumumab	Yes	9/24/2013	Postmarket BBW	Yes	Fatal PML reported
Obinutuzumab	Yes	11/1/2013	Postmarket BBW	NA	Fatal PML reported
Belimumab	No	4/1/2014	Postmarket warning	NA	Cases of PML systemic lupus erythematosus while taking other immunosuppressant
Vedolizumab	No	5/20/2014	Premarket warning	NA	Cases of PML reported with other integrin receptor antagonists
Ocrelizumab	No	3/28/2017	Premarket warning	NA	PML with other anti-CD20 antibodies and other multiple sclerosis treatments but no cases in ocrelizumab trials
Alemtuzumab	No	10/1/2017	Warning postmarket	NA	Nonfatal case of PML in postmarketing experience
Polatuzumab	No	6/10/2019	Warning premarket	NA	Has been reported after treatment

BBW = black box warning; NA = not applicable; PML = progressive multifocal leukoencephalopathy.

**Figure 2:**
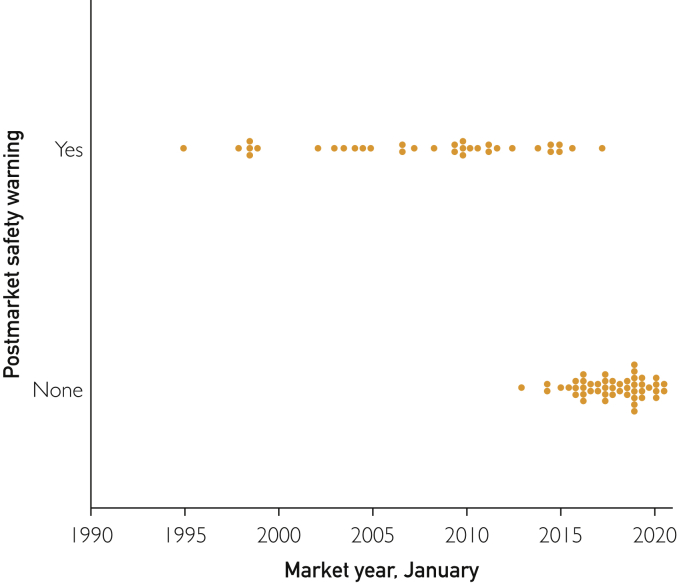
Initial postmarket safety warnings according to market year. Of the 83 monoclonal antibodies, 34 had a postmarket safety warning.

Among these 83 mAbs on the market, 34 had a previous postmarket safety event. Median time to the first overall postmarket safety events for mAbs that received a warning was 1.56 (IQR, 0.71-2.76) years. Postmarket safety warnings were present in 12 (63.2%) of the 19 mAbs with accelerated approval compared with 22 (34.4%) of 64 agents without accelerated approval (*P*=.03). Otherwise, postmarket safety warnings were not associated with priority review, breakthrough therapy, fast-track approval, orphan drug status, premarket BBW, or time from FDA submission to approval. We were unable to identify any mAb that had been on the market for longer than 8 years that had not received a postmarket safety event by the FDA (Figure 2). Median years on the market for mAbs with specific postmarket warnings were 16.13 (IQR, 9.77-19.81) compared with 3.36 (IQR, 1.63-5.64) years for agents without postmarket BBWs (*P*<.001). Median years on the market for mAbs with postmarket safety events were 10.72 (IQR, 5.85-16.90) compared with 2.56 (IQR, 1.40-4.03) for agents without postmarket safety events (*P*<.001).

## Discussion

The primary objective of the present study was to determine the potential risk for an emerging BBW that may occur after a patient’s therapy is initiated with an FDA-approved mAb. On average, specific BBWs emerged in the postapproval period at a rate of 3.4% (19/562) per year. Most postmarket BBWs were preceded by an FDA warning at a median time of 3.61 (IQR, 1.36-5.78) years. The emergence of a novel BBW not preceded by a specific FDA product label warning was 0.9% (5/562) annually. The Institute of Medicine has recommended consideration of the safety throughout the entire pre and post approval lifecycle of therapeutic agents.^17^ Risks for adverse effects exist for mAbs in the postmarket period and include the risk for a BBW. The potential for these risks should be reviewed with patients before the initiation of treatment.^18^

The numbers of postmarket BBWs and overall postmarket safety events were associated with the time that the mAbs were on the market. Various potential explanations are possible for this association. An increased number of patient-years when taking the treatment may have allowed adverse events that were not recognized in the relatively low number of patient-years required for phase 2 and phase 3 clinical trials before market approval. Reportedly, a learning curve has been required for the development and formulation of mAbs or the potentially related regulatory review, which included recognition of adverse events and subsequent advances in antibody development that may have reduced future risk.^19^ We suspect that a combination of these factors accounts for the association between the number of warnings and the time that an mAb was on the market.

Although the most common category of postmarket BBWs was related to infectious disease, only 1 of the 5 warnings not predicted by a specific product label warning was infectious in nature. Progressive multifocal leukoencephalopathy was first recognized as a result of mAb treatment when used in natalizumab-treated patients who had relapsing forms of multiple sclerosis and Crohn disease.^20^, ^21^, ^22^ Since that time, a vigilant assessment of mAbs appears to have been done on the potential for the adverse effect of PML. Safety warnings have preceded other BBWs for PML for all mAbs other than natalizumab. Cofactors for a risk for PML have been identified. The FDA has provided guidance for clinicians seeking to use these agents with optimal safety.

The need for the FDA and other regulatory bodies’ robust assessments and recommendations following the identification of PML as a potential adverse effect of specific mAbs serves as an example of a highly unexpected, potentially unpredictable sentinel event, which in the case of PML occurred initially with natalizumab.^23^ A review of mAbs with general warnings or BBWs to PML appears to reflect a continuous improvement process that has evolved over time as the FDA strives to promote identification of risks and thus promote safe use in patients requiring mAb treatment.

Specific postmarket BBWs were not associated with expedited forms of regulatory review (ie, priority review, accelerated approval, breakthrough therapy, fast-track approval, orphan drug status, or regulatory review time) or a premarket BBW in the present study. Overall, postmarket safety events were significantly associated with accelerated approval but not with other markers of expedited review or premarket BBW. An increase in postmarket safety events was associated previously with accelerated approval in a mixed cohort of pharmaceuticals and biological agents.^24^

The present report has limitations. The data were taken mainly from the massive FDA database of previous historical regulatory reviews, which may not have been complete for previously withdrawn agents and which had a number of broken links. In part because of these reasons, the study included only therapeutic mAbs that previously were FDA approved but were active on the market in March 2020. Thus, the study excluded muromonab-CD3, daclizumab, and efalizumab, which had been previously withdrawn from the market for safety concerns, and excluded vedolizumab, which was reportedly withdrawn from the market voluntarily. The overall number of mAbs was limited, which potentially could have impaired our ability to optimally characterize associations with specific risk categories. Inclusion of mAbs approved within the past 7 years may not have allowed sufficient time in the postmarket period to detect all new postmarket warnings that may occur with these agents over their therapeutic life cycle.

Despite these potential limitations, the present report has several distinct characteristics, including a focus on mAbs, which now make up a rapidly developing compilation of more than 80 FDA-approved therapeutic agents. This report details all specific postmarket BBWs while it recognizes that mAbs may contain more than 1 specific warning in a particular black box and that a particular agent may have received more than 1 specific postmarket BBW.

The present study observed that most BBWs were preceded by a general safety warning in a previous FDA label. This study’s estimate of BBWs that occurred in the postmarket period could be used as a starting point by a medical provider to initiate discussions to inform their patients about the possibility of yet-unforeseen adverse effects that could arise in those receiving mAbs. This article also shows the potential benefit of informing patients of the helpful National Library of Medicine website (https://dailymed.nlm.nih.gov). On this website, patients can review important information about the pros and cons of treatments that they and their clinicians are considering.

## Conclusion

During the past several years, an abundance of novel mAbs have undergone FDA approval. Postmarket warnings occur commonly with these agents. The BBWs have also occurred at an estimated rate of 3.4% per year. For FDA-approved mAbs, the rate of the emergence of a new BBW not preceded by a specific FDA product label warning may be less than 1% (5/562) per year. The FDA’s continued regulatory review and guidance are appreciated as clinicians seek to optimally identify and communicate specific risks and benefits of novel therapeutic mAbs for patients who have medical conditions for which previous effective or safe therapies have not been available.
